# Comparison of Chinese Broiler Production Systems in Economic Performance and Animal Welfare

**DOI:** 10.3390/ani10030491

**Published:** 2020-03-15

**Authors:** Qichang Chen, Helmut W. Saatkamp, Jan Cortenbach, Weidong Jin

**Affiliations:** 1 Department of Agriculture Economic and Management, Shenyang Agriculture University, Shenyang 100161, China; 2017200135@syau.edu.cn; 2 Business Economics Group, Wageningen University, Hollandseweg 1, 6706 KN Wageningen, The Netherlands; helmut.saatkamp@wur.nl; 3 Wellhope-De Heus Animal Nutrition B.V. Nr. 2 International Mansion 2, 9A-B, Information rd. 2, Beijing 100085, China; jcortenbach@deheus.com

**Keywords:** animal welfare, broiler production system, economic performance

## Abstract

**Simple Summary:**

There are three main rearing systems for white-feathered broilers in China. They are the net floor system (NFS), the normal cage system (NCS), and the high standard cage system (HCS). This study compared the relationship between economic benefit and animal welfare between these systems. The high economic input, high output, and high profit in these three different rearing systems. The welfare scores were 778.24 ± 29.45, 691.09 ± 32.97, and 669.82 ± 22.79, respectively. As white-feathered broiler production in China has developed, from the conventional system to the latest system, both cost and economic profit have increased while the welfare score has decreased. This study explains why the level of animal welfare in China’s white-feathered broiler production is not high at present, and why breeders do not wish to improve the level of animal welfare production.

**Abstract:**

Both proper animal welfare and economic benefit are important to the broiler industry, so it is better to consider these two factors together. The purpose of this study was to investigate the relationship between economic benefit and animal welfare in different production systems of white-feathered broilers in China. Based on the Welfare Quality Assessment (WQA) protocol for poultry, the authors compared and evaluated the results of the Welfare Quality model (WQM) and the deterministic model. The present study conducted welfare evaluations and investigations on 66 broiler chicken flocks on 52 farms in China. These flocks included three types: the net floor system (NFS), the normal cage system (NCS), and the high standard cage system (HCS). In terms of economy, the results were in line with high economic input, high output, and high profit. In terms of animal welfare assessment, the authors calculated the welfare scores per measure and the attributional WQ scores and WQ index scores of each production systems. The results showed that nine welfare measures from four welfare criteria presented different trends in the three production systems. WQ index scores were 778.24 ± 29.45, 691.09 ± 32.97, and 669.82 ± 22.79, respectively. According to Chow test results, significant differences were found between WQ index scores and total cost and profit (all *p* < 0.01). In conclusion, with the development of white-feathered broiler production in China, from the conventional system to the latest system, both cost and economic profit have been increased, but the welfare score has been decreased.

## 1. Introduction

In the past few decades and especially in recent years, as in many other countries, the public in China has become more concerned about animal welfare [1]. When compared to the laying hen and fattening pig industries, broiler production has the best short-term and mid-term prospects for developing animal-friendly market concepts [2]. 

In the past 40 years, the broiler industry has developed rapidly in China and its production is the second largest in the world after USA. In addition, the broiler industry in China has become the second largest animal husbandry after the pig industry, and the development of white-feathered broiler industry has been especially striking [3,4]. However, animal welfare and related research in the broiler industry are still at a low level in China [1]. Furthermore, most studies focus only on the concept and significance of animal welfare [5,6,7,8]. Excitingly, more studies have focused on consumer willingness to pay (WTP) and the actual level of welfare during the process of production in recent years. Nowadays, the Chinese middle-class has begun to be more concerned about animal welfare [9]. These results are similar to the research in many countries, such as Belgium, the Netherlands, and especially Brazil [10,11,12] where socio-demographic characteristics present the largest differences in consumer attitudes [13]. 

A comparison of perforated flooring (netting) systems and the conventional litter floor system in production performance and welfare showed that each system has its pros and cons [14]. Studies have proved that floor systems show advantages in production performance and welfare, however, in the field of white-feathered broilers, farmers still prefer the cage system instead of the flooring system [3,4]. Even so, the efficiency of broiler production in China still lags behind the highest levels in the world [15]. Therefore, it is valuable to evaluate the animal welfare and economic benefits of the white-feathered broilers production model in China, thus promoting the welfare self-evaluation and self-improvement of the production sector [16]. 

Nowadays, based on the Welfare Quality Assessment (WQA) protocol for poultry, more assessments of the welfare level of broiler production have been performed [17]. These studies have focused on a specific single measure in the welfare protocol, such as plumage condition [18], stunning [19], light intensity [20], gait score [21], and dark system [22]. In order to reduce bias and accurately calculate the overall Welfare Quality (WQ) index score for each system, Gocsik et al. (2016) used a weighted index based on WQA to relate system attributes to welfare measures [23,24]. 

Currently, yellow- and white-feathered broilers are mainly bred in China’s broiler industry. There are three main production systems for white-feathered broilers: the net floor system (NFS), the normal cage system (NCS), and the high standard cage system (HCS) [3,4]. The main objective of this paper is to compare and analyze the economic performance and animal welfare of the three systems. This thesis is structured in the following steps: in the first step, based on the survey data, a deterministic model was used to obtain economic performance; in the second step, the assessment data and Welfare Quality model were used to obtain welfare scores per measure, attributional WQ scores, and WQ index scores of each system; and in the third step, economic performance and WQ index scores were compared by using correlation analysis and the Chow test.

## 2. Materials and Methods 

This study included at least two aspects of data, economic performance and animal welfare assessment. Production data was collected on the farm by interviews. Some of the data were summarized from the delivery reports, which were sourced from the slaughterhouse. These included farm information, flock information, technical information, and cost information. Assessment data of animal welfare and production were collected at 66 flocks on 52 farms. The authors conducted an assessment of animal welfare of poultry in accordance with the guidance of the Animal Welfare Quality Broiler Assessment protocol [17]. A flock was defined as broiler chickens in a single coop at a particular farm depopulated on the same date [23]. In the NFS, chickens could walk back and forth when they were young, yet with low density and semi-confinement. In the NFS, almost all work was done manually, and the sample flock was eight in this report. The NFS has quickly been replaced by the NCS, but in the past few years, the NCS has been fully upgraded to the HCS. In the NCS and HCS, several chickens are isolated in a half-square-meter iron cage with high density and total confinement. The major difference between the NCS and HCS is whether they use the automatic feces-scraping equipment. As a result, the environmental condition of the NCS is poor, because the feces cannot be removed on time. In addition, the HCS is equipped with an automated feeding system and an automated adjustment system of ventilation and temperature control (NCS flocks, *n* = 20; HCS flocks, *n* = 38). Compared with the fully automated HCS, the NCS can be called semi-automatic. Therefore, the fundamental difference between these three systems lies in initial cost.

In general, the assessment was performed in accordance with the Welfare Quality Assessment [17]. Most of the measures could be assessed but some measures differed from the description in the assessment protocol. For example, mortality and cull rates are two different indicators used to assess the score of good health in the WQA. In this study, however, only mortality was used. Since the farmers did not record the number of broilers culled separately, only the total mortality rate was available for the entire data set. This treatment was the same as that of Ingrid’s study (2016) [23]. 

Due to inadequate data of flocks in the litter system in this study, the assessment of the litter system was cancelled. According to the protocol, the animals will initially be disturbed when the observers visit observation points, and then the response and expressive quality of their activity at the group level will be included in the assessment. However, it is impossible to observe and evaluate broilers in dim, restricted, and high-density cages in which the density is more than fifty kilograms per square meter (Table S1). Thus, the assessment of qualitative behaviors was cancelled in this research as well. According to the WQ protocol, the percentage of broilers with ascites, septicemia, hepatitis, pericarditis, and abscesses should be recorded at the slaughterhouse. However, only the total percentage of rejected broiler chickens and the number of small broiler chickens are recorded in Chinese slaughterhouses [23].

Costs and benefits were calculated for all three systems using a deterministic model. The model was redesigned based on the research of Gocsik [24]. Variable costs, fixed costs, and total cost were calculated in Chinese currency (CNY) per delivered broiler. Based on the deterministic model, together with the survey data on the farm, and the delivery report back from the slaughterhouse, the authors could get all the production costs as well as the return of the broiler flocks, such as the price of day-age chickens, feed cost, health cost, heating cost, water and electricity cost, catching chicken cost, feed transportation cost, chicken transportation cost, labor cost, infrastructure construction, and inventory investment.

**Figure 1:**
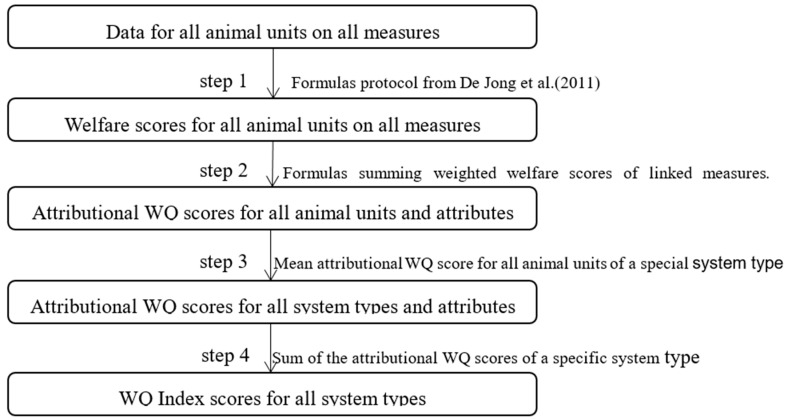
Overview of steps for calculation of the Welfare Quality (WQ) index score.

A stepwise approach, modified from Gocsik et al. [24] (Figure 1), was used to obtain a WQ index score for each production system. First, the welfare score per measure for each flock was assessed (Step 1 in Figure 1). Calculations were carried out according to the WQ protocol. Second, for each flock, the attributional WQ score was calculated per system attribute by multiplying each welfare score by its weight associated with a given attribute and subsequently by summing up the weighted scores obtained per attribute (Step 2 in Figure 1):

$$\mathrm{WQ}\;-{\;A}_{jk\;}=\sum^{\;}\left(w_{ik}\times {\;x}_{ij}\right)$$

where WQ − A_jk_ is the attributional WQ score for each flock and system attribute k, w_ik_ is the weight of the link between welfare measure i and system attribute k (the value of w_ik_ is very crucial for the next calculation), and x_ij_ is the welfare score for welfare measure i and flock j. The weight matrix was from the research of Gocsik et al. (2016) [24] (Table S1). 

Third, the attributional WQ scores per production system were calculated as the mean of the attributional WQ scores of the flocks of a given production system (Step 3 in Figure 1):

$$\mathrm{WQ}\;-\;A_{km}=\sum \;(\mathrm{WQ}\;-\;A_{jk})/n$$

where WQ − A_km_ is the attributional WQ score for production system m and n is the number of farms of production system m. 

Finally, the overall WQ index score per production system was calculated by adding up the mean attributional WQ scores for each production system (Step 4 in Figure 1):

$$\mathrm{WQ}\;-\;I_{m}=\sum \;\mathrm{WQ}\;-A_{km}$$

where WQ − I_m_ is the WQ index score for production system m. It is important to note that the overall WQ index score can be calculated in an alternative way, that is, by calculating the mean of the welfare scores for each flock and summing up the mean welfare score per welfare measure for each production system. All the data was collected based on the animal welfare questionnaire.

In order to examine the impact of total cost or benefit on Welfare Quality scores in the three different production systems, it was necessary to test the coefficient differences of three subsamples. The Chow test and Fisher’s permutation test are common methods used to test coefficient difference between groups after grouping regression [25,26]. The authors used the Chow test as a reference and introduced cross-terms for testing significance of the coefficient and the intercept. In this study, two dummy variables, d1i and d2i, were introduced. If one sample belonged to NFS, then d1i = 0 and d2i = 0; if one sample belonged to NCS, then d1i = 1 and d2i = 0; and if one sample belonged to HCS, then d1i = 0 and d2i = 1. The model of this study was set as follows:

$$WQ_{i}=\propto +\mu d1_{i}+vd2_{i}+\beta var_{i}+\pi \;\left(var_{i}\times d1_{i}\right)+\varphi \;\left(var_{i}\times d2_{i}\right)+\varepsilon_{i}$$

where, ∝, β, μ, v, π, and φ are the parameters. ε_i_ is the common error term of this formula, var_i_ is the independent variable, which is the be total costs and profit in this article, d1_i_ and d2_i_ are two dummy variables that mean the different systems in this research, (var_i_ × d1_i_) and (var_i_ × d2_i_) are two cross terms of system and total costs (profit), WQ_i_ is the Welfare Quality score of each flock and i is a flock in this research, and it varies from 1 to 66.

In NFS, d1i = 0, d2i = 0, the formula can be expressed as follows:

$$WQ_{i}=\propto +\beta var_{i}+\varepsilon_{i}$$

In NCS, d1i = 1, d2i = 0, the formula can be expressed as follows:

$$WQ_{i}=\propto +\;\mu \;+\;(\beta \;+\;\pi )\;{\mathrm{var}}_{i}+\varepsilon_{i}$$

In HCS, d1i = 0, d2i = 1, the formula can be expressed as follows:

$$WQ_{i}=\propto +\;v\;+\left(\beta +\varphi \right)\;var_{i}+\varepsilon_{i}$$

## 3. Results

### 3.1. Technical Performance

The technical performance of the three production systems is listed in Table 1. Significant differences were found between the NFS and the cage system, but the values of the one-way test showed no obvious differences between the NCS and the HCS. Among these indexes, there were significant differences in terms of density, mortality, and rounds per year. The cage systems had the highest density with the lowest mortality. At the same time, the farmers with the cage systems can make, on average, nearly six rounds of products per year, but those with NFS can make only 4.5 rounds per year because more time is needed to clean up the barn for removal of the worse environmental conditions, and sometimes the farmers with NFS have to wait for good market conditions. The Europe production index (EPI) is one of the most critical indicators in livestock production. Under the same production conditions, a large value of EPI usually indicates a better profit. In this study, the value of EPI was 323.7 (NFS), 368.4 (NCS), and 377.8 (HCS), respectively, which means that HCS had the best profit. In addition, analysis of variance showed that there was a significant difference between the NFS and the cage rearing system, but no significant differences were found between the two kinds of cage systems. As shown in Table 1, the value of EPI varied considerably in the NFS, however, in the HCS, the value of EPI was more stable.

### 3.2. Economic Performance

- Total cost: Table 1 shows the input and output in the different production systems. For each delivered broiler, the total costs were increased from NFS to HCS and were 26.51 CNY, 26.74 CNY, and 27.81 CNY, respectively. However, the total costs for per delivery kilogram decreased and were 10.48 CNY, 9.67 CNY, and 9.62 CNY respectively. The total costs can be divided into two parts: the variable costs and the fixed costs. The variable costs accounted for a larger proportion of total costs than the fixed costs. The lowest proportion was 89.3% in NFS, and HCS had the highest proportion, which was 93.9% (Table 1).
- Variable cost and fixed cost: Feed price was the main factor contributing to the increase in total costs, which accounted for around 60%–70%, and the one-day chick price accounted for 17.3% to 18.2% of the total costs (Table 1). Feed price and chick price together accounted for 79.5% (NFS), 84.0% (NCS), and 84.9% (HCS) of the total costs, respectively. Meanwhile, health care costs are also important for raising broilers. The authors found that NFS had the highest health care fee, which was approximately 50% more than that of the cage systems. One of the main differences between the three systems was the labor cost. As shown in Table 1, the hiring labor cost in the HCS was 0.305 CNY per broiler chicken, which was almost two times higher than that of the NCS, and the hiring labor cost in the NFS was zero CNY. In contrast, the cost of own labor in the NFS was the highest, almost 2.4 times higher than that of the NCS. The cost of the own labor was close to zero CNY in the HCS (Table 1).
- Return and profit: NFS had the lowest, and HCS had the highest return of the three systems. If the authors considered the total cost, the profit in NFS would be negative (-2.47 CNY per broiler chicken). The profit of the HCS was 37% more than that of NCS, which was 1.37 CNY per broiler chicken (Table 1).

### 3.3. Broiler Animal Welfare

The stepwise approach model was used to resolve the research question of what was the WQ index score of each production system. As shown in Figure 1, the mean welfare score per measures, all the mean attributional WQ scores from each flock, the mean attribution WQ score of each system, and WQ index score were counted.

- Welfare score per measure: Fourteen measures were cited in the present research model, and they were divided into four categories: good feeding, good housing, good health, and appropriate behavior [17]. Measurements of emaciation and thirst indicate good feeding levels. Measurements of cleanliness, dust, panting, and stocking indicate the level of good housing. Lameness, hock burn (HB), foot pad dermatitis (FPD), breast blister (BB), mortality, and ascites indicate the level of good health. The outdoor and avoidance distance test (ADT) indicates the level of appropriate behavior. Nine out of fourteen measures showed significant differences between the three systems (*p* < 0.05). The measures of cleanliness, panting, FPD, lameness and the ADT did not show significant differences. The most obvious difference between NFS, NCS, and HCS was stocking density. In NFS, the score was 56.25, while in NCS and HCS, the scores were 0.36 and 0.89, respectively (Table 2).
- Attributional WQ scores: The mean attributional WQ scores of all flocks as well as each system type were showed in Table 3. In terms of all the flocks, the mean WQ scores of broiler-types was 220.92. As a percentage of the population it was 32.4%, and was the major contributor. The WQ score of the dark length period was 138.9, and the proportion was 20.4%, which was the second contributor. The third contributor was the outdoor access, for which the attributional WQ score was 115.92 and the proportion was 17%. The fourth contributor was the stocking density, for which attributional WQ score was 114.82, and the proportion was 16.9%. These four attributes accounted for 86.7% of the total WQ index score. The assessment results in Table 3 also show the significant difference in density, dark length period, and flock size (*p* < 0.05). The stocking density had the most significant difference in attributes. The score for NFS was 164.94, accounting for 21.2%, which was more prominent than that NCS (112.65) and HCS (105.43). However, the welfare score in density measurement could be considered as the main reason for the advantages of NFS accounting for a 61% increment from NCS to NFS, and a 55% increment from HCS to NFS (Table 3).
- WQ index score: The animal WQ index scores are also given in Table 3 according to production system. The WQ index score sharply decreased when shifting from NFS (778.24) to NCS (691.09) or HCS (669.82) and significant differences were found between all systems (*p* < 0.05). The standard errors of the WQ index scores were 29.45 (NFS), 32.97 (NCS), and 27.79 (HCS) respectively indicating that NCS had the broadest range of variation.

### 3.4. Correlation Analysis

**Figure 2:**
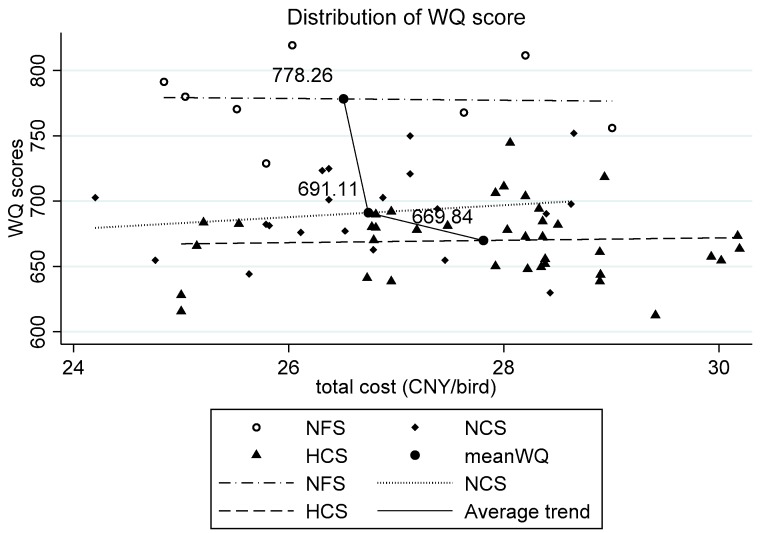
Distribution of WQ scores based on cost of per delivered broiler. NFS = normal floor system, NCS = normal cage system, and HCS = high standard cage system.

**Figure 3:**
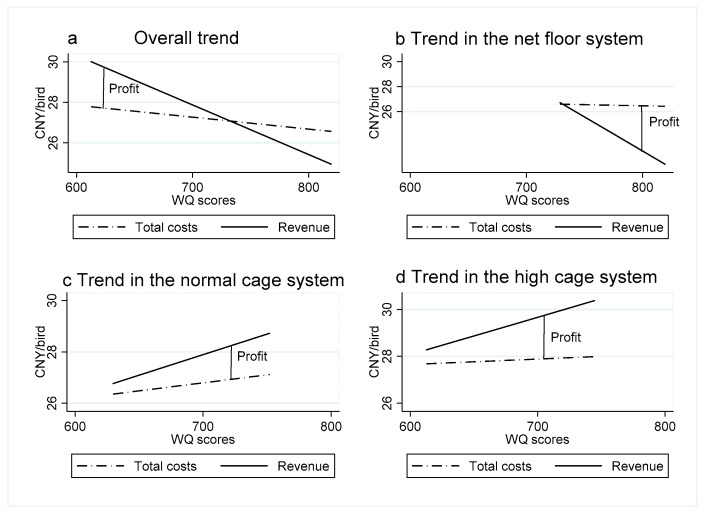
Correlation between the total cost, revenue profit and the WQ per system type. (**a**) Overall trend; (**b**) Trend in the net floor system; (**c**) Trend in the normal cage system; (**d**) Trend in the high cage system.

- Distribution and trends: Figure 2 shows the distribution and trends based on the total costs of the different systems. Points in three different patterns showed the distribution of the WQ score of each system. NFS was on the top, and had only a small fraction of intersection with NCS, but no intersection with HCS. That means the flocks in NFS mostly had a higher WQ score. The areas of NCS and HCS were mostly overlapped. According to Figure 2, the fitting straight line of the three systems was at the different heights. The broken line, which was drawn by the mean values of these three systems, showed that there was a downward trend from NFS to HCS (Figure 2).
- Correlation between the economic performance and AW: The correlation between the total costs, the revenue, the profit, and the WQ scores are shown in Figure 3. These four diagrams show the correlation in all the samples and each system. The semi-dotted line represents the relationship between total costs and the WQ score. The solid line represents the relationship between total revenue and the WQ score. The gap represents the profit, which was equal to the total revenue minus the total costs.
- When the animal WQ score increased, total costs and revenue continued to decline, and profits continued to fall until they reach zero, when the losses were getting greater and greater. Similar trends were shown in Figure 3a,b. However, when the animal WQ score was relatively low, the objective data of profits were obtained only from a small number of flocks in NFS. As shown in Figure 3c,d, when the WQ score increased, both the revenue and the total costs increased, and the profit grew as well.
- Chow test results: To test significant differences in the effect of WQ score on total costs and profit among the three systems, the authors used the Chow test method twice for reference and tested by introducing cross items to test whether there were significant differences in WQ coefficients between the three groups. Table 4 presents the Chow test results in stata12.0 analysis; in this table, d1and d2 are two dummy variables and total-cost-d1 and total-cost-d2 are the interaction items. The *p* value of all the variables were significant at the level of 0.01, which means the three systems showed significant differences in the impact of the total costs on WQ score. d3 and d4 were the other two dummy variables, profitd3 and profitd4 were the interaction items. The Chow test showed that the impact of WQ score on profit was significantly different between NFS, NCS, and HCS (*p* < 0.01).

## 4. Discussion

The objective of this study was to compare the economic performance and animal welfare of three systems of white-feathered broilers production in China based on the Welfare Quality model and correlation analysis. Recently, after comparing five production systems in Europe, Gocsik et al. (2016) concluded that the middle-market systems could be attractive for farmers due to their high cost-efficiency and the flexibility to revert to the conventional system [24]. A comparison of total confinement and semi-confinement systems for broiler chickens in Brazil also confirmed that the birds subjected to the semi-confined system had better opportunity to express their natural behavior and explore the external environment to the module of production, leveraging the animal welfare [27].

When considering economic performance, the cost of broiler production in China is higher than that in some other countries, such as the U.S. and Europe [15,24]. In the current study, NFS showed the lowest cost and the lowest profit of the three systems (Table 1). That may be due to the lowest delivery weight and the highest disease and mortality during the same feeding period, plus the highest health costs. Variable costs are the most important cost among the three systems. In addition, feed costs and chicken prices account for the largest proportion of total costs, which could partially explain why NFS systems in China have been replaced so quickly by NCS and HCS. The present results are consistent with many other studies [24,28]. 

From the welfare score of each measure, there were no stable trends in the 14 measures. This result is consistent with Gocsik’s conclusion [24] and confirms the feeling of farmers who own cage systems. These farmers do not believe that cage systems have the lowest welfare level because the chickens have a higher performance in some measures. At the same time, they disagree with the idea that appropriate behavior is equally as important as good feeding, good housing, and good health [29]. In The present research, the assessment results of cleanliness and FPD were close to zero, because all the farmers adopted net rearing broiler chickens, which can separate the broiler from feces. In the process of welfare assessment, the authors also found that two chicken houses with similar conditions may have considerable differences in welfare score per measure. One of the main reasons may be due to the management level and attitude of managers. Many researchers have started to consider the impact of management on animal welfare levels [30,31]. Further research is therefore required to analyze management factors in WQA.

In evaluating the attributional WQ score, the present results are consistent with Gocsik’s viewpoint. The sum of the four attributes accounted for 86.7% of the total attribute score, compared with 82% in her research. The high attributional WQ scores for these four attributes can be explained by the relatively high welfare scores on the welfare measures and the high weights [24]. The present results were slightly different from those of her research in terms of numerical value or ranking but show a general convergence. Broiler type is the most important contributor. Density is very important too, and these two factors are the main sources of difference. This finding is in common with other studies [32,33]. It is well documented that correct stocking density is necessary to obtain optimal broiler performance [34]. Higher density brings higher yield but low welfare [35,36]. In the current system in China, the stocking density of NFS is lower than the optimal stocking density, however, NCS and HCS are higher than the optimal stocking density.

In terms of WQ index score, NFS showed the highest result (778.24), with NCS (691.09) and HCS (669.82) welfare score showing a downward trend. This is consistent with the evaluation of the lower welfare level of the cage system in some other literature. Shields (2013) believes that there are some advantages to controlling broilers in cages, however, this will eventually be replaced due to the requirements of animal welfare and food safety [37]. A feature of the present study was to give weight to each measure so that the scores of different criteria and attributes could be pulled together and compared. The final attributional WQ score and the WQ index score had the same proportion as Gocsik’s results [24], but because the selected indicators were not the same, the final score was only compared in the three broiler systems in China. In future, this method will also be applied to more broiler production systems, especially more complex production systems including yellow-feathered broilers. This research only considered the assessment information on the farm, but it should also cover the production period on the farm and the period at the end of life, including transport and slaughter [17]. Many studies have suggested that animal welfare can be improved in terms of post-hatch brooding temperature at hatcheries, stunning broilers in the slaughterhouse, and supplying organic acid during transportation [38], and measures for slaughter [39]. These are also areas of future research with the hope of systematically improving animal Welfare Quality index scores.

On the correlation analysis of economic performance and welfare score, the results showed a contradictory trend. In the overall samples of three systems (NFS, CNS, and HCS), WQ index score, total costs, and profit showed a negative correlation. The relationship between NFS and the whole sample keep consistent change relationship. The reason may have been due to the mortality rate of NFS being too high. On the one hand, this will reduce the welfare score [24], but on the other hand, it will also reduce the density at the time of evaluation [17], however, it will eventually increase the overall welfare score. There may also be another reason that the current broiler market does not have a product price classification [1] although it has higher welfare, but cannot have higher product prices. The internal performance of NCS and HCS showed a positive correlation between welfare score, total costs, and profit. This result is consistent with the mainstream view that welfare, costs, and profit are positively correlated [24]. This is because the increase in investment will enrich the environment and then improve welfare. Welfare improvements can also reduce health costs [40,41,42,43]. Based on previous studies, the authors propose making full use of available technology to improve the environment, such as lighting and cage furnishing [20], and eventually improve animal welfare. The government can subsidize animal welfare products to compensate for the increase in market prices and strengthen the control of the label system [1].

Overall, the present study showed that the welfare score in the newest system was low, but the economic benefit was good. Meanwhile, it also showed that greater economic benefits can be achieved by increasing investment, enriching the environment, and improving the welfare scores in NCS and NHS. This conclusion is consistent with Marian Stamp Dawkins’ view that conflicts can be resolved and financial benefits can reinforce rather than replace good animal welfare [10], resulting in a win–win situation for poultry welfare and production [40].

At present, the results of animal welfare research from different perspectives are quite different. Fouad (2008) thought cages were not recommended as a management system for rearing broilers from both the economic and welfare points of view [44]. Welfare scores depend on the variety, feed, housing, and preferences of different consumers [33]. Different subjects, consumers, producers, and governments show different preferences [11,29,45,46]. Differences in preferences constitute a dilemma in government regulation [47]. That is why animal welfare has always been a hot topic of discussion. Economy and welfare should be considered together but due to many economic or practical constraints they will never be implemented on commercial farms and thus never benefit animals [43]. Commercialization and marketization are good ideas to promote animal welfare [48]. In any case, improving animal welfare levels and product safety is an inevitable trend. The authors need to consider the economic and welfare issues together and make full use of commoditization and market-oriented means to promote the welfare development of the poultry industry.

## 5. Conclusions

According to the comparison of three production systems, HCS paid the highest production costs and obtained the highest return. In contrast, NFS paid the lowest production cost and obtained the lowest return. In terms of variable costs, both the variable cost as a percentage of total costs and total cost of HCS was the highest in three systems. Regarding fixed costs, both the absolute value of fixed costs and fixed cost as a percentage of total costs of NFS were the highest in three systems. In short, HCS has the highest profit and efficiency; NFS has the lowest profit and efficiency.

In the current three white-feathered broilers production systems, NFS had the highest WQ score, followed by NCS, while the HCS had the lowest WQ score. Welfare and system attributes have different trends in both the welfare score and the attribute animal WQ score. The system attributes contributing most to AW were broiler type, length of the dark period, outdoor access, and stocking density. On the average of the three systems, the welfare scores of these four attributes accounted for nearly 86% of the total welfare level.

As far as the relationship between animal welfare and production costs is concerned, NFS had the lowest production costs and the highest welfare level, while the high standard cage system had the highest production costs and the lowest welfare score. In term of the correlation between the WQ index score with the factors of NFS, the total costs and the profit decreased while the WQ index score was increasing. In contrast, the total costs, and the profit increased when the WQ index score increased. Significant differences were found between all the three systems.

In conclusion, the WQ index scores of NCS and HCS were lower than that of NFS, but its economic profit was higher than that of NFS. At the same time, intra-group changes investments and WQ index scores and profits of NCS and HCS maintained an upward trend.

## Figures and Tables

**Table 1:** Technical inputs, costs, and profits per delivered broiler.

Item	Unit	Production System					
		NFS ^3^		NCS ^3^		HCS ^3^	
		Mean	SD	Mean	SD	Mean	SD
Technical variables							
Length of growth period	day	43.5 ^a^	2.93	44.8 ^b^	0.83	44.8 ^b^	0.65
Daily growth	g	58.3 ^a^	3.74	64.1 ^b^	3.39	64.5 ^b^	2.62
Delivery weight	kg	2.53 ^a^	0.13	2.87 ^b^	0.15	2.89 ^b^	0.12
Density	kg/m^2^	26.5 ^a^	4.8	53.5 ^b^	5.99	51.4 ^b^	5.05
FCR ^1^		1.69 ^a^	0.13	1.68 ^a^	0.032	1.65 ^a^	0.03
Mortality	%	6.92 ^a^	2.65	3.79 ^b^	1.24	3.26 ^b^	1.03
Rounds	#/year	4.61 ^a^	0.74	5.9 ^b^	0.31	6 ^b^	0.16
EPI ^2^	#	323.69 ^a^	47.93	368.41 ^b^	25.19	377.77 ^b^	19.63
		mean	%	mean	%	mean	%
Variable costs		23.68 ^a^	89.30%	24.95 ^a^	6.70%	26.12 ^b^	93.90%
1-d chick ^4^	CNY/bird	4.58 ^a^	17.30%	4.83 ^b^	18.10%	5.05 ^c^	18.20%
Feed	CNY/bird	16.49 ^a^	62.20%	17.61 ^a^	65.90%	18.56 ^b^	66.70%
Health care	CNY/bird	1.52 ^a^	5.70%	1.1 ^b^	4.10%	0.96 ^c^	3.50%
Heating	CNY/bird	0.38 ^a^	1.40%	0.48 ^b^	1.80%	0.48 ^b^	1.70%
Electricity	CNY/bird	0.18 ^a^	0.70%	0.18 ^a^	0.70%	0.18 ^a^	0.60%
Transport (chicks)	CNY/bird	0.37 ^a^	1.40%	0.49 ^a^	1.80%	0.40 ^b^	1.40%
General cost	CNY/bird	0.198 ^a^	0.70%	0.212 ^a^	0.80%	0.214 ^a^	0.80%
Transport (feed)	CNY/bird	0.20 ^a^	0.80%	0.25 ^a^	0.90%	0.21 ^a^	0.80%
Labor (hire)	CNY/bird	0.000 ^a^	0.00%	0.13 ^a^	0.50%	0.305 ^b^	1.10%
Fixed costs	CNY/bird	2.83 ^a^	10.70%	1.78 ^b^	6.70%	1.70 ^b^	6.10%
Labor (own)	CNY/bird	1.12 ^a^	4.20%	0.47 ^b^	1.80%	0.05 ^c^	0.20%
Build	CNY/bird	0.70 ^a^	2.60%	0.36 ^b^	1.30%	0.50 ^b^	1.80%
Inventory	CNY/bird	1.01 ^a^	3.80%	0.96 ^b^	3.6%	1.15 ^b^	4.10%
Total cost	CNY/bird	26.51 ^a^	100.00%	26.74 ^a^	100.0%	27.81 ^b^	100.00%
Return	CNY/bird	24.04 ^a^	90.70%	27.75 ^b^	103.80%	29.19 ^c^	104.90%
Profit	CNY/bird	−2.47 ^a^	−9.30%	1.01 ^b^	3.80%	1.37 ^b^	4.90%

^a–c^ One-way test: values with different superscripts indicate significant differences between systems. (*p* < 0.05). ^1^ FCR = feed conversion ratio, ^2^ EPI = Europe Production Index, EPI = delivery weight × (1-mortality) × 10000/(FCR × length growth period), ^3^ NFS = normal floor system, NCS = normal cage system, and HCS = high standard cage system, ^4^ 1-d chick = The price of one-day-old chicks.

**Table 2:** Mean welfare score per measure per system type.

System	NFS ^5^		NCS ^5^		HCS ^5^	
	Mean	SD	Mean	SD	Mean	SD
Emaciated	61.46 ^a^	14.00	79.76 ^b^	12.03	88.70 ^c^	12.48
Thirst	99.74 ^a^	13.92	73.04 ^b^	9.15	76.93 ^b^	6.92
Cleanliness	99.42 ^a^	0.00	99.42 ^a^	0.00	99.42 ^a^	0.00
Dust	65.50 ^a^	13.36	85.70 ^b^	10.77	81.89 ^b^	11.66
Panting	69.25 ^a^	23.06	76.80 ^a^	16.94	68.39 ^a^	19.39
Stocking density	56.25 ^a^	9.00	0.36 ^b^	1.61	0.89 ^b^	4.12
Lameness	81.98	0.00	81.98	0.00	86.16	0.00
HB ^1^	76.20 ^a^	16.15	75.34 ^a^	18.44	55.28 ^b^	13.34
FPD ^2^	100 ^a^	0.00	98.94 ^a^	3.22	99.06 ^a^	2.42
BB ^3^	56.65	0.00	33.48	0.00	33.48	0.00
Mortality	60.51 ^a^	15.18	87.14 ^b^	17.35	89.51 ^b^	13.94
Ascites	71.50	0.00	100	0.00	100	0.00
Outdoor	13.00	0.00	13.00	0.00	13.00	0.00
ATD ^4^	93.42 ^a^	3.31	24.63 ^b^	0.00	24.63 ^b^	0.00

^a–c^ One-way test: values with different superscripts indicate significant differences between systems. (*p* < 0.05). ^1^ HB = hock burn, ^2^ FPD = foot pad dermatitis, ^3^ BB = breast blister, ^4^ ATD = avoidance distance test, ^5^ NFS = normal floor system, NCS = normal cage system, and HCS = high standard cage system.

**Table 3:** Mean attributional WQ scores and WQ index scores per system type.

System Attributes	Production System Attributional WQ Scores											
	NFS ^1^			NCS ^1^			HCS ^1^			Total		
	Mean	SD	%	Mean	SD	%	Mean	SD	%	Mean	SD	%
A1. Broiler type	217.59 ^a^	10.74	28	225.62 ^a^	11.39	32.6	219.15 ^a^	10.98	32.7	220.92	11.36	32.4
A2. Length growth	33.46 ^a^	3.23	4.3	33.28 ^a^	3.69	4.8	30.20 ^b^	2.67	4.5	31.52	3.4	4.6
A3. Weight at delivery	15.24 ^a^	3.23	2	15.07 ^a^	3.69	2.2	11.06 ^b^	2.67	1.7	12.77	3.63	1.9
A4. Enrichment	9.11	0	1.2	9.11	0	1.3	9.57	0	1.4	9.37	0.23	1.4
A5. %Grain in feed	25.02 ^a^	0	3.2	24.74 ^a^	0.8	3.6	24.77 ^a^	0.6	3.7	24.79	0.63	3.7
A6. Stocking density	164.94 ^a^	20.28	21.2	112.65 ^b^	11.05	16.3	105.43 ^b^	10.12	15.7	114.82	22.37	16.9
A7. Outdoor access	107.90 ^a^	5.06	13.9	116.49 ^b^	5.85	16.9	117.31 ^b^	4.73	17.5	115.92	5.88	17
A8. Daylight	9.11	0	1.2	9.11	0	1.3	9.57	0	1.4	9.38	0.23	1.4
A9. Length of dark period	164.75 ^a^	6.02	21.2	136.82 ^b^	6.48	19.8	134.55 ^b^	5.92	20.1	138.9	11.44	20.4
A10. Flock size	31.14 ^a^	1.1	4	8.21 ^b^	0	1.2	8.21 b	0	1.2	10.99	7.54	1.6
Total WQ index score	778.24 ^a^	29.45	100	691.09 ^b^	32.97	100	669.82 ^c^	27.79	1	681.4	45.25	100

^a–c^ One-way test: values with different superscripts indicate significant differences between systems. (*p* < 0.05). ^1^ NFS = normal floor system, NCS = normal cage system, and HCS = high standard cage system.

**Table 4:** Chow test analysis of WQ index score, total cost, and profit.

WQ	Coef.	Std.Err.	*t*	*p* > t	[95%Conf.Interval]	
Total cost	29.27	0.451	64.9	0.000 ***	28.368	30.172
d1 ^1^	568.931	170.503	3.34	0.001 ***	227.989	909.873
d2 ^1^	644.561	110.42	5.84	0.000 ***	423.763	865.359
Total cost d1	−24.701	6.387	−3.87	0.000 ***	−37.472	−11.931
Total cost d2	−28.362	3.991	−7.11	0.000 ***	−36.342	−20.382
Profit	−166.017	19.9	−8.34	0.0000 ***	−205.81	−126.224
d3 ^2^	675.224	69.67	9.69	0.0000 ***	535.918	814.529
d4 ^2^	652.614	57.13	11.42	0.0000 ***	538.379	766.849
Profit d3	181.66	57.38	3.17	0.0020 ***	66.922	296.398
Profit d4	178.559	40.074	4.46	0.0000 ***	98.426	258.692

*** denote *p* < 0.01, d1 and d2 are two dummy variables in model 1, if d1_i_ = 0 and d2_i_ = 0, system = normal floor system (NFS), if d1_i_ = 1 and d2_i_ = 0, system = normal cage system (NCS), and if d1_i_ = 0 and d2_i_ = 1, system = high standard cage system (HCS). ^2^ d3 and d4 are two dummy variables in model 2, if d3_i_ = 0 and d4_i_ = 0, system = normal floor system (NFS), if d3_i_ = 1 and d4_i_ = 0, system = normal cage system (NCS), and if d3_i_ = 0 and d4_i_ = 1, system = high standard cage system (HCS).

## Supplementary Materials

The following are available online at https://www.mdpi.com/2076-2615/10/3/491/s1, Table S1: Matrix showing weights between AW attributes and welfare measures.
