# Supplementary material for: Comparison of Chinese Broiler Production Systems in Economic Performance and Animal Welfare
Source: Animals (Basel). 2020 Mar 15;10(3):491. doi: 10.3390/ani10030491 (PMC7142404; doi:10.3390/ani10030491)
Supplement: Supplementary file 1 [file animals-10-00491-s001.pdf]

Table S1. Matrix showing weights between AW attributes and welfare measures.

	system attributes(k)									
Welfare measures(i)	k1.Broiler type	k2.Length growth period	k3.Weight at delivery	k4. Enrichment	k5.%Grain in feed	k6.Stocking density	k7.Outdoor access	k8. Daylight	k9.Length dark period	k10.Flock size
i1.Plumage cleanliness						0.5	0.5			
i2.Litter quality		0.2				0.4		0.2	0.2	
i3.Panting	0.5					0.5				
i4.Stocking density						1				
i5.Lameness	0.22	0.22		0.11		0.11		0.11	0.22	
i6.Hock burn	0.2	0.2	0.2			0.2			0.2	
i7.Foot pad dermatitis	0.25				0.25		0.25		0.25	
i8.Breast blister	1									
i9.On-farm mortality	0.33						0.33		0.33	
i10.Ascites	0.67								0.33	
i11.Free range							1			
i12.ADT [1]									0.67	0.33
i13.QBA [2]				0.33		0.17		0.17	0.17	0.17

[1]ADT= Avoidance distance test. [2] QBA=Qualitative behavioral assessment.

In the second step of the Welfare Quality Model, the value of wik is very crucial for the next calculation. Table 1 of the matrix is from the research of Gocsik et al (2016). There are two steps at least to work out the table — the first step is to find out the link between AW attributes and welfare measures. In the second steps, weights have to be given to all linkages to create allocation formulas for calculating the WQ index scores. All the information comes from the literature study. The welfare measures used in this literature study are based on the Welfare Quality Assessment protocol for poultry (Welfare Quality®,2009). A matrix between the welfare measures and the system attributes was built at first. If some pieces of literature were found to support a linkage between measures and attributions, then it would be marked. If not, there would be empty in the matrix.

According to other studies, some connections were found to be more profound than others. For some welfare measures, this tendency was not significant, and therefore, all linkages with these specific welfare measures obtained equal weights. The weighing procedure included several steps and was implemented as follows. First, for each welfare measure, the linkages were evaluated on their relative importance. When a linkage was least profound, it obtained an important factor of one. In case of a stronger relation between a welfare measure and a system attribute, the linkage obtained an important factor of two, and in case of a direct relationship between a welfare measure and a system attribute, the linkage received an important factor of three. Therefore, the relative importance increased with an increasing factor value.

For the establishment of allocation formulas, the sum of the weights of all linkages for each welfare measure had to be equal to one. Therefore, the important factors were transformed into weights. An overview of the weights between system attributes and welfare measures is given in Table S1 [1].

References

1 Gocsik, E.; Brooshooft, S.D.; De Jong, I.C.; Saatkamp, H.W. Cost-efficiency of animal welfare in broiler production systems: A pilot study using the Welfare Quality® assessment protocol. Agric. Syst. 2016, 146, 55–69.
